# Sleep and Sleeplessness

**Published:** 1887-04-09

**Authors:** 


### SLEEP AND SLEEPLESSNESS.

This is an age of hurry and bustle. The struggle to live becomes harder day by day, because the population is increasing so rapidly. Amongst the lower orders early marriages promote and aggravate the evils attendant upon this condition of affairs. Immature and ill-developed parents produce pigmy offspring, and so the race tends to degenerate, especially in cities. The introduction of the telegraph and of steam, the increased facilities for traffic of all kinds, and the aggregation of immense populations in narrow areas, all tend to oppress the individual, and to interfere with his repose and rest. Hence the necessity for, and the value of, sleep were never greater than at present, because probably they were never more difficult to procure, to an extent equal to the demands upon the system of most brain-workers, and very many others too. Cervantes, long ago, and wisely, called down blessings on him who invented sleep. He declared sleep to be the mantle that covers all human thoughts; the food that appeases hunger; the drink that quenches thirst; the fire that warms cold; the cold that moderates heat; and lastly, the general coin that purchases all things, the balance and weight that equals the shepherd with the king, and the simple with the wise. He giveth his beloved sleep, has been the utterance of many a thankful heart in times of exceptional weariness and fatigue.

Tired nature's sweet restorer, balmy sleep, with fresh air and pure water, are the common inheritance of princes and peasants, and of healthy persons in all parts of the world. Yet man, with his ambitions, his strivings after power, his injustices, his necessities and his indiscretions, has wilfully done much to banish sleep from his own and from his neighbours' eyes; is doing so at the present moment, and will probably continue to do so until the end of the world. Still, it is well to remember that sleep is the natural inheritance of the healthy, the best friend of frail humanity, and, like all other friends, best estimated in its loss. It is life's nurse sent from heaven to create us anew day by day; and the loss of sleep, if continued, must be followed by the loss of health, and end in the loss of life itself.

Sleeplessness being dangerous to life, it is important to ask, what are its chief causes? Overwork, excess in diet, in tobacco, in alcohol, neglect of ordinary precautions in the management of oneself, in people who are otherwise healthy and free from disease, are the fruitful producers of insomnia. It may be remembered that in former days, when the English law enacted that people condemned to death should be prevented from sleeping, the majority of the convicts died raving maniacs. Dr. Forbes Winslow has pointed this out, and has shown that those who are starved to death become insane, because the brain is not nourished, and they cannot sleep. Non-recuperation from absence of rest and want of nourishment causes sleeplessness, and means madness in the end. Those who think most, who do most brain-work, require most sleep. Time, which some people declare themselves to have "saved" from necessary sleep, means in the end the infallible destruction of mind and body. Give, then, yourself, your children, your servants, give all who are under you the fullest amount of sleep they will take, by compelling them to go to bed at some regular early hour and to rise in the morning the moment they awake. Such is Dr. Winslow's advice, and he backs it by the declaration that within a fortnight, under such conditions, nature, with almost the regularity of the rising sun, will unloose the bonds of sleep the moment enough repose has been secured for the wants of the system. How much sleep anyone requires is a question each must decide for himself by experiment and practice. Under such conditions of life sleep would seldom or never fail the healthy, and the household would benefit in temper, in regularity, in the quality of the work done, and in the addition to the family chest, which the combined efforts of its members must produce under such advantageous circumstances.

Let us now return for a moment to a consideration of the causes and cure of sleeplessness. Prolonged mental strain in all its various phases is a common cause of sleeplessness. The student preparing for an examination, the friend depressed by the sudden death of a loved relative, the young professional man broken down by the continual pressure of new disappointments and accumulating anxieties, are all cases of acute or continued mental strain, which may result in sleeplessness. The subjects of this form of insomnia are mostly men of nervous temperament, that is, men whose temperament presents a distinct type and outward form of manners, habits, and tendencies. The aim in all these cases must be to prevent the sleeplessness by removing its causes, instead of pursuing the easier, but illogical and precarious, course of permitting it to continue, and trusting to suppress one of its effects by medicine. When a man cannot sleep because he works his brain too much, the remedy is cessation from or greatly lessening labour. Real work is, however, not often the cause of sleeplessness, but worry, anxiety, harass, which bring unrest. It is not work that wears, but worry.

To tell a busy man, with the absorbing claims of a vast business, that he must leave his office and give up work for weeks, or months, will not cure his insomnia, but rather tend to aggravate his symptoms. One of the most eminent physicians of the day, dealing with such a case in the course of his practice, ordered his patient to take two beefsteaks and a quart bottle of India pale ale for luncheon every day for a fortnight, and then to see him again. At the end of the fortnight the patient protested he could not get through his work if the treatment was continued; but continued it was for six weeks, with the result that the patient's work was reduced to reasonable proportions, and a complete cure was effected, because the patient readily assented to take a holiday, involving change of scene and complete rest, as the result of his physician's diplomacy.

In the case of smokers, excessive smoking, with the progressive advance from mild to the strongest tobacco, means in most cases sleeplessness in the end. Even slight alteration or excess in the daily quantity of tobacco may produce unrest, followed by sleeplessness. In the same way a little more than the usual quantity of wine after dinner, or even the usual quantity of some unusual wine, will produce a like effect.

Doctors often meet with cases of sleeplessness in which alcohol alone is the cause of the wakefulness. A man may even pride himself upon his moderate use of stimulants, and yet be made sleepless by the amount of alcohol he consumes. In such cases a cessation or a reduction in the tobacco or alcohol will usually prove an effectual remedy. In the case of old persons and others, where sleeplessness becomes almost a habit, a simple and often effectual cure is to have an Etna by the bedside, which will keep milk, or beef-tea, or soup, warm throughout the night. When sleep forsakes such persons, then the taking of a little warm food will often prove effectual in producing healthful and sustaining sleep. We have not space to go further into this interesting question, and it will be seen that we have purposely refrained from recommending the prescription of medicines, because our experience teaches us that anything in the way of narcotic treatment should generally be avoided if possible.

